# Summer Eating

**Published:** 1869-07

**Authors:** 


### SUMMER EATING.

People are too apt to render themselves liable to disease by means of the food taken into the stomach.

During hot weather, like the present, you should remember that the system does not require so much fuel, in the form of food, as you have been in the habit of taking. Very little, or no meat at all should be eaten, as the temperature of the body is greatly increased through its instrumentality, while the opposite effect should be maintained. Health is best preserved in those who live upon a mixed diet. It is not a good plan to have the same meats and the same vegetables every day for a week or two—change them frequently. Vegetables are necessary to healthfulness, in this climate, without them we should soon become diseased with scurvey. The vegetables possessing the highest proportion of flesh-forming principle, are the bean, pea, beet, potato, &c.

Two meals a day is best in hot weather. Breakfast about nine in the morning, and have your dinner about four in the afternoon. You then escape the greatest heat of the day, when it is absolutely injurious to eat plentifully.

Milk should not be drunk at meals during this season, it will render you bilious.. In fact, very little drink of any kind should be indulged in at meals. A wine-glass of sour-wine—pure grape juice—is the most health-producing drink at meals ; it assists digestion, when the secretions in the stomach are not plentifully supplied, as in cool weather.

Give more of your attention to your diet this summer, and learn how to improve your health thereby.

POISONOUS SOCKS.—The dye used in some of the gorgeous red socks displayed in the windows of metropolitan hosiers, exercises a very deleterious influence upon the skins of the wearers, producing irritation and eruption, and finally actual sores.

—There is a Doctor for every 1000 inhabitants in Paris.

—Knife handles and fine-tooth combs are now made from potato pulp by a chemical process.

